# Characterization of multivalent complexes formed in the presence of more than one conventional antibody to terminal complement component C5

**DOI:** 10.1371/journal.pone.0284502

**Published:** 2023-04-20

**Authors:** Josh Cone, Lida Kimmel, Yuchun Zhang, Krista Johnson, Douglas Sheridan, Paul Tamburini

**Affiliations:** 1 Alexion, AstraZeneca Rare Disease, New Haven, CT, United States of America; 2 Alexion Pharmaceuticals, Inc., New Haven, CT, United States of America; The University of Kansas, UNITED STATES

## Abstract

This study sought to understand the nature of the immune complexes that could be formed when a patient is exposed simultaneously to two different anti-complement component 5 (C5) antibodies, such as in patients converting from one bivalent, noncompetitive, C5-binding monoclonal antibody to another. Size exclusion chromatography (SEC) in combination with multiangle light scattering was used to assess the potential formation of multivalent complexes among eculizumab, C5, and each of two other anti-C5 bivalent antibodies, TPP-2799 or TP-3544, respectively having the same sequence as either crovalimab or pozelimab currently undergoing clinical trials. Each of these two antibodies bound C5 noncompetitively with eculizumab. In phosphate-buffered saline (PBS), C5-eculizumab in the absence of other antibodies measured <500 kDa; however, inclusion of other antibodies at levels ranging from equimolar and up to a fivefold excess over eculizumab and C5 yielded a series of complexes with some >1500 kDa in size, consistent with incorporation of multiple antibodies and C5 molecules. A similar pattern of complexes was also observed when fluorescently labeled eculizumab and either of the other two antibodies were spiked into human plasma, based on SEC monitored by fluorescence detection. A detailed characterization of the pharmacodynamic and pharmacokinetic properties of such complexes is warranted, as is the incorporation of mitigation processes to avoid their formation in patients converting from one bivalent, noncompetitive, C5-binding monoclonal antibody to another.

## Introduction

The properties of circulating immune complexes formed with target antigens should be considered when developing a monoclonal antibody (mAb). Valency of such complexes can affect clearance, effector function, and uptake by phagocytic cells. Many conventional therapeutic antibodies bind monovalent targets, circumnavigating the potential to form polyvalent immune complexes between target and antibody. However, significant care and consideration must be given to situations in which a recipient is exposed to more than one antibody that can bind to a monovalent target at different sites. Depending on precise epitopes, such conditions can potentially lead to the formation of multivalent complexes with unexpected *in vivo* properties. For example, this could arise when a patient who had previously received a bivalent antibody to complement component 5 (C5) is switched to receiving a second bivalent antibody targeting a different site on C5, while finite levels of the first antibody remain in circulation to ensure continuous pharmacodynamic suppression.

Eculizumab is a humanized mAb that is directed against C5 and which has been transformative for patients with paroxysmal nocturnal hemoglobinuria (PNH), atypical hemolytic uremic syndrome (aHUS), refractory generalized myasthenia gravis (gMG) and aquaporin-4-IgG-positive neuromyelitis optica spectrum disorder [1–5]. Ravulizumab (ALXN1210) [6], was subsequently developed through further engineering of eculizumab and is the first long-acting, C5 inhibiting antibody effective in patients with PNH [7], aHUS [8] and adult patients with gMG who are anti-acetyl choline receptor antibody positive [9]. Ravulizumab is expected to bind to the same epitope on C5 as eculizumab in macroglobulin domain (MG7) [10] and has a longer duration of action and mean terminal half-life than eculizumab, as well as a different pharmacodynamic and clinical profile.

The effectiveness of these therapies has advanced understanding of the role of the complement system in disease. Other therapeutic antibodies targeting the complement system have been developed, with some targeting different epitopes on C5 from the one targeted by eculizumab and ravulizumab. Such antibodies include crovalimab [11] and pozelimab [12], which are in clinical development [13, 14] as potential interventions for complement-related disorders including PNH.

In one study, patients with PNH receiving eculizumab were switched to crovalimab. Complexes among crovalimab, C5, and eculizumab, which are referred to as drug-target-drug complexes (DTDCs), were observed in all 19 patients of the COMPOSER phase 1/2, part 3 study who were switched from eculizumab to crovalimab, with two patients exhibiting mild-to-moderate vasculitic skin reactions that were considered to be drug-related and possibly DTDC-related [13].

Therefore, we sought to precisely define the size and stoichiometry of multivalent complexes in compositions containing C5 and antibodies binding C5 at different non-overlapping sites *in vitro*. The findings in our study may have implications for patients who are receiving these other anti-C5 antibodies while continuing to have detectable levels of eculizumab in circulation.

## Materials and methods

### Source of antibodies

Eculizumab Drug Substance was used throughout this study. Ravulizumab Drug Substance was used in all studies to characterize immune complexes by size exclusion HPLC. Laboratory grade ravulizumab was highly purified by chromatography over a HiTrap Protein A HP column (GE Healthcare, cat# 17-0403-01). C5-binding mAb N19-8 [15], included as a control in some experiments, was purified from the parent hybridoma. MAb TPP-2799, was generated in-house using the sequences of a Chugai Pharmaceutical mAb designated as RO7112689 (also known as crovalimab) and described in CAS Registry Number 1917321-26-6. MAb TPP-3544 was generated in-house based on the sequences described in US Patent #10633434 (heavy chain, SEQ ID NO 353; light chain, SEQ ID NO 354; Regeneron Pharmaceuticals, Inc.) [16], later known as REGN3918 and more recently named as pozelimab. Sequences were synthesized and cloned separately into a mammalian expression vector (GeneArt Gene Synthesis, Thermo Fisher Scientific, Waltham MA), and transiently expressed in an Expi293 Expression System kit. The respective mAbs were purified using MabSelect SuRe^TM^ chromatography (GE Healthcare, cat# 11-0034-95)).

### Protein labeling

Eculizumab was labeled with Alexa Fluor 488 (Thermo Fisher Scientific, cat# A10235) according to the manufacturer’s specifications. For bio-layer interferometry (BLI) studies, eculizumab (2 mg/mL) in phosphate-buffered saline (PBS) was biotinylated by mixing with 10 mM EZ-Link NHS-PEG4-Biotin (No-Weigh Format, Thermo Fisher Scientific, cat. #21329, lot #SI254541), achieving a 10-fold molar excess of biotin and incubated for 60 minutes at room temperature followed by incubation on ice for 60 minutes. Excess unbound biotin was removed with Zeba Spin Desalting Columns (Thermo Fisher Scientific, cat. # 89882, lot #RD232015). The concentration of biotinylated eculizumab was then measured with NanoDrop 1000 spectrophotometer (Thermo Fisher Scientific), wherein the absorbance measured at lambda 280 was used to calculate the protein concentration. Biotinylation of eculizumab by this method does not significantly affect the affinity for C5 based on comparative BLI analysis on two separate occasions.

### Binding of antibodies to C5

The competition between anti-C5 mAbs was evaluated using BLI on an OctetRed instrument (Sartorius). To assess the degree to which the anti-C5 antibodies TPP-2799, TPP-3544, N19-8, and ravulizumab, competed with eculizumab for binding to C5, biotinylated eculizumab (2 μg/mL) in PBS containing 0.2 mg/mL bovine serum albumin and 0.004% (v/v) Tween 20, and hereafter referred to as Kinetics Buffer, was loaded onto a streptavidin-coated biosensor (Pall ForteBio, division of Pall Life Sciences, Waltham, MA) for 300 seconds. The biosensor was then washed in Kinetics Buffer for 120 seconds before being exposed to purified human C5 (hC5; Complement Technology, Tyler TX, cat. #A120) at 50 nM in Kinetics Buffer for 300 seconds, or Kinetics Buffer devoid of C5 as a control. After washing with Kinetics Buffer for an additional 100 seconds, the biosensor was exposed to either TPP-2799, TPP-3544, eculizumab, N19-8, or ravulizumab at 100 nM in Kinetics Buffer for 300 seconds. The presence or absence of an additional signal associated with this last step showed whether the second antibody added could bind with C5 noncompetitively or competitively with eculizumab, respectively.

### Blockade of hemolysis by anti-C5 antibodies

Chicken red blood cells (cRBCs) in 0.4 mL Alsever’s solution (Lampire Biological Laboratories, Pipersville PA, cat. #7201403), were washed four times with 1 mL GVB^++^ buffer (0.1% gelatin, 5 mM Veronal, 145 mL NaCl, 0.025% NaN3 pH 7.3 containing 0.15 mM CaCl_2_ and 0.5 mM MgCl_2_ (Complement Technology, cat. #B100) at 4°C and resuspended to 5x10^7^ cells/mL in 6 mL of GVB^++^. A polyclonal anti-cRBC antibody (Rockland Immunochemicals, Pottstown PA, cat. #103–4139) was added to the cRBCs to achieve a final concentration of 120 μg/mL before incubating for 15 minutes on ice. After washing twice with GVB^++^, the cells were resuspended to a final 3.6 mL in GVB^++^. Purified anti-C5 antibodies were separately serially diluted (1:1) with GVB^++^, and 50 μL aliquots were mixed with 50 μL aliquots of 40% (v/v) healthy human serum (complement preserved from Complement Technology, cat. #NHS) and incubated at room temperature for about 30 minutes. Sensitized cRBCs (30 μL per well, 2.5x10^6^ cells) were then added to the anti-C5 antibody/sera incubations and further incubated at 37°C for 30 minutes. The plates were then centrifuged at 1700×g for 3 minutes and 80 μL of the supernatants were transferred to a flat-bottom 96-well plate. The samples were read at 415 nm using a SpectraMax i3x Multi-Mode Microplate Reader (Molecular Devices, San Jose CA) and the percentage of hemolysis was calculated as below.


$$percentage\;of\;hemolysis=\frac{OD415\;sample-OD415\;0\%\;lysis}{OD415\;100\%\;lysis-OD415\;0\%\;lysis}\times \;100$$

Abbreviation: OD = optical density.

The 100% lysis was assessed in incubations without anti-C5 antibody supplemented with 0.5% NP-40 Surfact-Amps Detergent Solution (Thermo Fisher Scientific, cat #85124). The 0% lysis was assessed in incubations without anti-C5 antibody supplemented with 20 mM ethylenediaminetetraacetic acid. Hemolysis (%) was plotted against the concentration of antibody present in 20% (v/v) serum.

### Identification and characterization of immune complexes

To determine the masses of complexes, purified eculizumab, hC5, and either TPP-2799 or TPP-3544 were incubated together in varying molar ratios in PBS for approximately 30 minutes at room temperature. Samples were placed at 2‒8°C in a refrigerated autoinjector for high-performance liquid chromatography (1260 LC System, Agilent, Santa Clara, CA, USA) and applied (45 μL/injection) in sequence to a 4.6×300 mm Bio SEC-5 Column, 5 μm 1000 A (Agilent, cat. #5190–2538) complete with upstream in-line 0.2 μm filter and previously equilibrated and run using PBS as mobile phase at 0.3 mL/minute. The column Vt = 4.2 mL (experimentally determined) and Vo = approx. 2 mL (estimate). Eluent was monitored at absorbance 280 nm, and an in-line multiangle light scattering (MALS) detector (Treos-II, Wyatt Technology, Santa Barbara, CA, USA) with a differential refractive index detector (Optilab T-rEX, Wyatt Technology) was used to quantify molecular masses. To evaluate the potential for such complexes to form in human plasma, eculizumab was replaced with Alexa Fluor 488-labeled eculizumab, and the incubations were performed in at least 80% (v/v) final human plasma (BioIVT, Westbury NY) with 35 μL injections into the size exclusion chromatography columns (SEC) column. The same chromatography method described above for samples diluted in PBS was used. The column was equilibrated and run in PBS and chromatography was monitored with in-line fluorescence detection using 1260 FLD Spectra fluorescence detector (Agilent, cat. #G1321B), in which elution was monitored with excitation at 490 nm and emission at 525 nm.

## Results

### Recombinantly expressed anti-C5 mAbs TPP-2799 and TPP-3544 are functionally active

**Fig 1:**
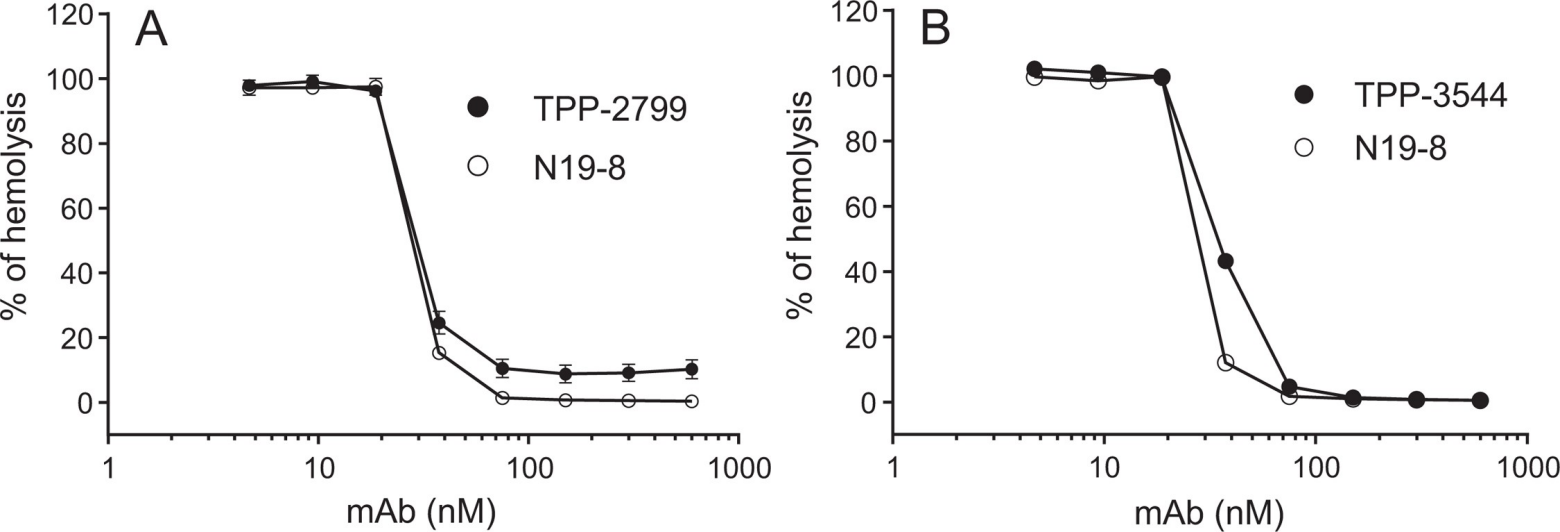
Recombinant anti-C5 mAbs TPP-2799 and TPP-3544 inhibit classical-pathway hemolysis. Anti-C5 mAbs TPP-2799 (closed circles in panel A) and TPP-3544 (closed circles in panel B) showed potent and dose-dependent inhibition of classical-pathway hemolysis, similarly to the C5-binding mAb N19-8 control (open circles in panels A and B). Abbreviations: C5 = complement component 5; mAb = monoclonal antibody.

TPP-2799 and TPP-3544 were expressed in Expi293 cells and were highly purified. Both antibodies showed dose-dependent inhibition of classical-pathway hemolysis of antibody-sensitized cRBCs in human serum (Fig 1), with half minimal inhibitory concentration (IC_50_) values of 30 and 35 nM for TPP-2799 and TPP-3544, respectively. The C5-binding mAb N19-8 [15] was also highly potent, with IC_50_ values of 34 and 35 nM in separate experiments. Given that these experiments were performed with 20% (v/v) final serum, the concentration of C5 would be expected to be approximately 80 nM, based on a literature value for the concentration of hC5 in serum of 75 μg/mL [17] or approximately 400 nM. Therefore, these IC_50_ values are in the expected range for tight-binding antibodies.

### Anti-C5 mAbs TPP-2799 and TPP-3544 bind to a different site on C5 than the site bound by eculizumab

**Fig 2:**
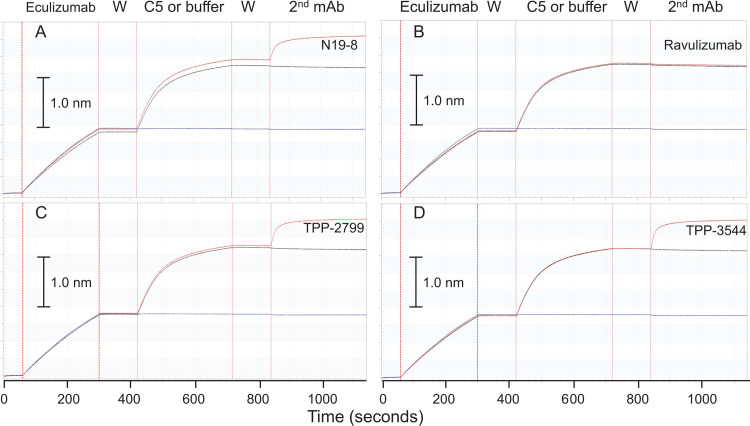
Recombinant anti-C5 mAbs TPP-2799 and TPP-3544 bind to hC5 noncompetitively with eculizumab. Biotinylated eculizumab was captured onto a streptavidin-coated biosensor probe, washed (W), and treated with either purified hC5 (red and black traces) or buffer (blue traces). Following an additional wash, probes were treated with either eculizumab (black traces), buffer (blue traces), or one of the following mAbs (red traces): (A) N19-8, (B) laboratory grade ravulizumab, (C) TPP-2799, (D) TPP-3544. The scale bar shows the deflection corresponding to a biosensor signal of 1.0 nm. Abbreviations: C5 = complement component 5; mAb = monoclonal antibody; W = washed.

Bio-layer interferometry on an Octet instrument was used to explore the degree of overlap among the hC5-binding sites of TPP-2799 and TPP-3544 and the hC5-binding site for eculizumab (Fig 2). Complexes formed on the streptavidin-coated biosensor tips between biotin-labeled eculizumab and C5 could subsequently bind to either TPP-2799 or TPP-3544 in a C5-dependent fashion, as evidenced by robust additional Octet signals. This behavior was similar to that of N19-8, which binds to a different site on C5 to than that bound by eculizumab [18]. In contrast, neither eculizumab nor ravulizumab bound to eculizumab–C5 complexes formed on the biosensor surface, because the eculizumab-targeted binding site was already occupied by the biotinylated eculizumab used to capture C5.

These data collectively showed that both TPP-2799 and TPP-3544 were functional and bound tightly to hC5 at sites that were different from the site bound by eculizumab; they also indicated that both TPP-2799 and TPP-3544 formed stable ternary complexes in the presence of eculizumab and hC5. Based on these findings, additional experiments were performed to determine whether mixtures of eculizumab, C5, and either TPP-2799 or TPP-3544 could form larger polyvalent structures, a theoretical possibility given that each antibody has two binding sites for hC5.

### Anti-C5 mAbs TPP-2799 and TPP-3544 induced the formation of high molecular weight complexes in the presence of hC5 and eculizumab

SEC with a column providing resolution between 50 and 7500 kDa was used to resolve and to characterize the complexes formed among various ratios of eculizumab, C5 and either TPP-2799 or TPP-3544. C5 was maintained at a concentration of 400 nM roughly equivalent to that of human blood. An eculizumab concentration of 100 μg/mL (700 nM) was selected to approximate the threshold blood concentration in humans, resulting in complete terminal complement inhibition [19]. Any switch from eculizumab to an alternative anti-C5 antibody would have to occur when the concentration of eculizumab is at or above this value to maintain continuous pharmacologic suppression. The concentrations of either TPP-2799 or TPP-3544 were varied, ranging from equimolar with eculizumab to a fivefold excess over eculizumab.

**Fig 3:**
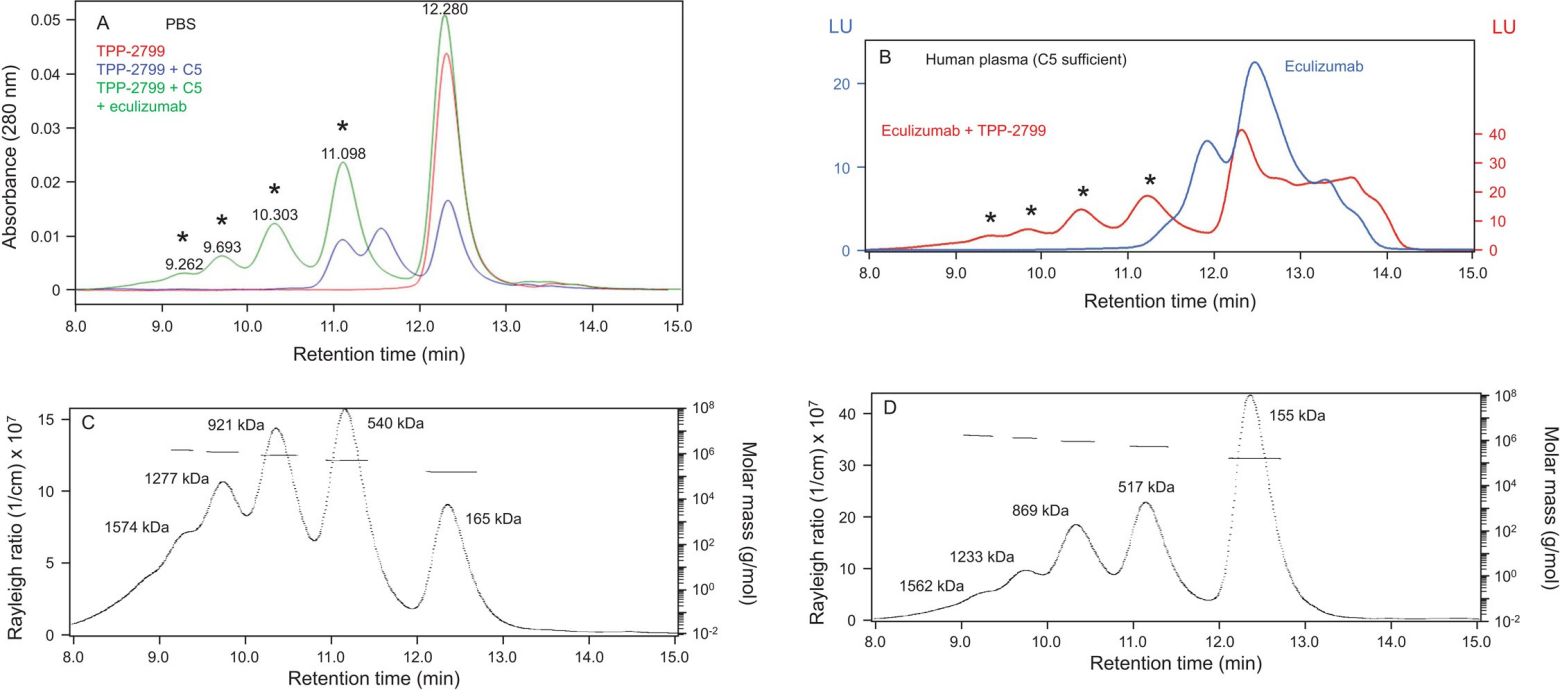
TPP-2799 induces the formation of very large complexes in the presence of eculizumab and C5. (A) TPP-2799 (700 nM) alone, in the presence of C5 (400 nM), or in the presence of C5 (400 nM) and eculizumab (700 nM) in PBS. Large, early-eluting peaks observed in the presence of TPP-2799, C5 and eculizumab are denoted with asterisks. (B) Reconstitution of Alexa Fluor 488-eculizumab (700 nM) in 80% human plasma in the absence or presence of TPP-2799 (700 nM). Early, eluting peaks observed in the presence of both the labeled eculizumab and TPP-2799 are denoted with asterisks. Data were plotted to normalize total chromatogram fluorescence area under the curve between the two treatment groups. (C) Rayleigh ratios and molar masses of complexes formed among TPP-2799 (700 nM), C5 (400 nM), and eculizumab (700 nM) in PBS. (D) Rayleigh ratios and molar masses of complexes formed among TPP-2799 (3500 nM), C5 (400 nM), and eculizumab (700 nM) in PBS. Abbreviations: C5 = complement component 5; LU = luminescence units; PBS = phosphate-buffered saline.

TPP-2799 alone migrated as a single peak (Fig 3A) with a MALS mass of 154 kDa (S1A Fig) and formed two additional species in the presence of C5 (Fig 3A) with masses of 400 and 516 kDa (S1B Fig), corresponding to the expected 1:1 and 1:2 TPP-2799:C5 complexes, given that C5 alone had a MALS mass of 205 kDa (S1C Fig). However, reconstitution of TPP-2799 with equimolar eculizumab plus C5 resulted in the formation of a series of large, early-eluting peaks (Fig 3A). Early-eluting complexes with highly similar retention times were also observed when TPP-2799 was reconstituted with equimolar labeled eculizumab in 80% human plasma, but not when TPP-2799 was absent (Fig 3B). In some cases, complexes formed among TPP-2799, C5, and eculizumab in PBS were greater than 1.5 million Da in MALS mass irrespective of whether reconstitution was with a concentration of TPP-2799 that was equimolar to (Fig 3C) or in fivefold molar excess over that of eculizumab (Fig 3D). Based on the known masses of C5 and IgG antibodies, the sizes of these complexes were in close agreement with those that would result from the incorporation of up to four molecules of C5 and five molecules of antibody (Table 1).

**Table 1:** Complexes formed among C5, eculizumab and either TPP-2799 or TPP-3544 at equimolar concentration with eculizumab vary in size and contain multiple copies of C5 and/or antibody.

	Eculizumab:TPP-2799 1:1		Eculizumab:TPP-3544 1:1	
Hypothetical complex	Predicted size (kDa)^a^	Observed size (kDa)^b^	Predicted size (kDa)^a^	Observed size (kDa)^c^
Free antibody A1 or A2	150	165	150	167
A1-C5-A2	535	540	539	569
A2-C5-A1-C5-A2	905	921	911	963
A1-C5-A2-C5-A1-C5-A2	1275	1277	1283	1314
A2-C5-A1-C5-A2-C5-A1-C5-A2	1645	1574	1655	1788

^a^Predicted sizes calculated using an observed free C5 MALS mass of 205 kDa (S1C Fig) together with observed sizes for monovalent free antibody of 165 kDa for composition with TPP-2799 and 167 kDa for compositions with TPP-3544.

^b^Masses taken from Fig 3C.

^c^Masses taken from Fig 4C.

Abbreviations: C5 = complement component 5; MALS = multiangle light scattering.

**Fig 4:**
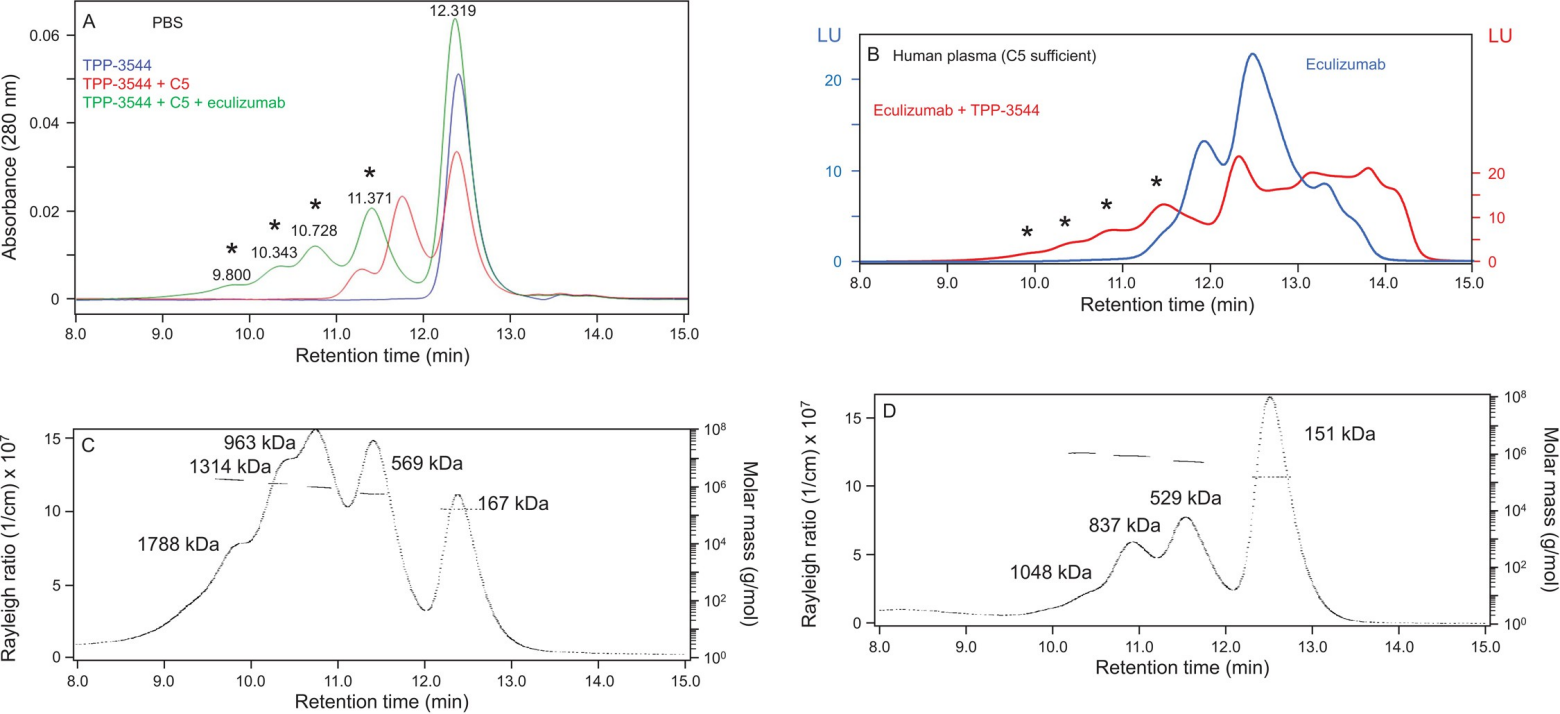
TPP-3544 induces the formation of very large complexes in the presence of eculizumab and C5. (A) TPP-3544 (700 nM) alone, in the presence of C5 (400 nM), or in the presence of C5 (400 nM) and eculizumab (700 nM) in PBS. Large, early-eluting peaks observed in the presence of TPP-3544, C5, and eculizumab are denoted with asterisks. (B) Reconstitution of Alexa Fluor 488-eculizumab (700 nM) in 80% human plasma in the absence or presence of TPP-3544 (700 nM). Early-eluting peaks observed in the presence of both the labeled eculizumab and TPP-3544 are denoted with asterisks. Data were plotted to normalize total chromatogram fluorescence area under the curve between the two treatment groups. (C) Rayleigh ratios and molar masses of complexes formed among TPP-3544 (700 nM), C5 (400 nM), and eculizumab (700 nM) in PBS. (D) Rayleigh ratios and molar masses of complexes formed among TPP-3544 (3500 nM), C5 (400 nM), and eculizumab (700 nM) in PBS. Abbreviations: C5 = complement component 5; LU = luminescence units; PBS = phosphate-buffered saline.

Similar results were obtained with TPP-3544 (Fig 4). TPP-3544 alone migrated as a single peak (Fig 4A) with MALS mass of 153 kDa (S2A Fig) and formed two additional species in the presence of C5 with masses of 382 and 563 kDA, corresponding to 1:1 and 1:2 TPP-3544:C5, respectively (S2B Fig). Reconstitution of TPP-3544 with eculizumab plus C5 resulted in the formation of a series of large, early-eluting peaks (Fig 4A). Early-eluting peaks with similar retention times were also observed when TPP-3544 was reconstituted with labeled eculizumab in 80% human plasma, but not when TPP-3544 was absent (Fig 4B). In some cases, as with TPP-2799, complexes formed among TPP-3544, C5 and eculizumab in PBS were greater than 1.5 million daltons in MALS mass (Fig 4C) and remained large even when TPP-3544 was at 3500 nM, a fivefold excess over eculizumab (Fig 4D). As with TPP-2799, the sizes of these complexes were in close agreement with those that would result from incorporation of up to four molecules of C5 and five molecules of antibody (Table 1).

**Fig 5:**
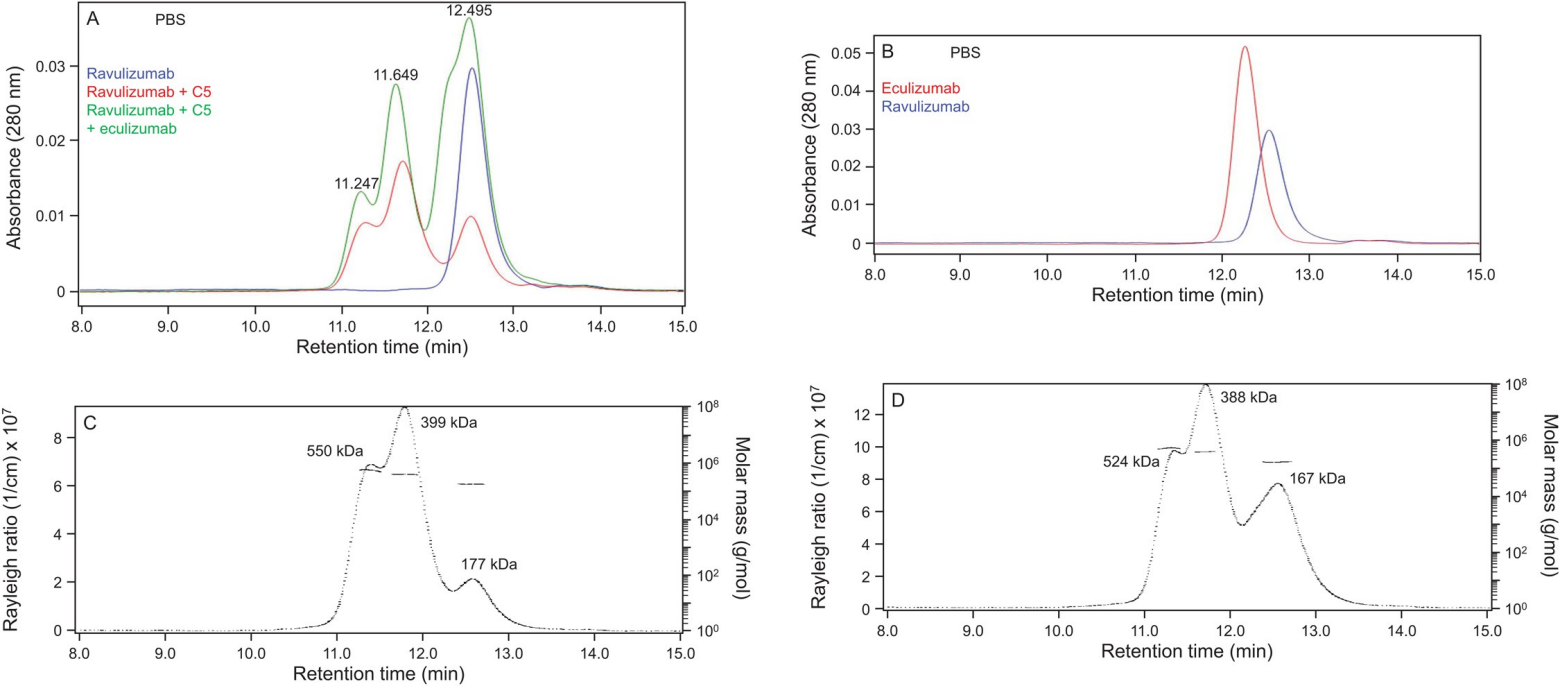
Ravulizumab forms only 1:1 and 1:2 complexes with C5 both in the absence and presence of eculizumab. (A) Ravulizumab alone (700 nM) migrated as a single peak but, in the presence of C5 (400 nM) or C5 (400 nM) plus eculizumab (700 nM), two additional early-eluting peaks were also observed. The last peak to elute in the composition of C5 plus eculizumab and ravulizumab in the region where free antibody elutes exhibited a shoulder. (B) Elution of free eculizumab and ravulizumab had slightly different retention times, explaining the shoulder in Fig 5A. (C) Rayleigh ratios and molar masses of complexes formed between ravulizumab (700 nM) and C5 (400 nM) in PBS. (D) Rayleigh ratios and molar masses of complexes formed in compositions comprising ravulizumab (700 nM), C5 (400 nM), and eculizumab (700 nM) in PBS. Abbreviations: C5 = complement component 5; PBS = phosphate-buffered saline.

The corresponding experiment was performed using compositions comprising ravulizumab, C5, and eculizumab (Fig 5). Ravulizumab alone migrated in PBS as a single peak (Fig 5A) with a MALS mass of 166 kDa (S3 Fig). As with compositions of TPP-2799 or TPP-3544 with C5, two additional, early-eluting peaks were also observed in compositions of ravulizumab plus C5, likely corresponding to 1:1 and 1:2 ravulizumab:C5 complexes (Fig 5A). However, in contrast to the results obtained with TPP-2799 and TPP-3544, compositions comprising ravulizumab, C5, and eculizumab did not produce a series of early-eluting peaks. Instead, peaks corresponding to the formation of 1:1 and 1:2 mAb:C5 complexes continued to be observed. Interestingly, a shoulder was also observed on the HPLC peak corresponding to free antibody (Fig 5A) which was explained by the fact that free ravulizumab and free eculizumab exhibit different retention times (Fig 5B), possibly due to differences in hydrophobic or electrostatic secondary interactions with the column stationary phase. Regardless, this does not affect the interpretation of the data showing a lack of complexes larger than the 1:1 and 1:2 mAb-C5 complexes. MALS analyses of compositions comprising ravulizumab and C5 (Fig 5C) yielded masses of 399 and 550 kDa, whereas MALS analyses of compositions of ravulizumab plus C5 and eculizumab yielded masses of 388 and 524 kDa (Fig 5D), which were close to those expected for 1:1 and 1:2 ravulizumab:C5 complexes.

## Discussion

This study explored the sizes of the complexes formed between hC5 and mixtures of anti-C5 mAbs using a combination of SEC and MALS. The recombinant anti-C5 mAbs TPP-2799 and TPP-3544 used in these studies have the same sequences as crovalimab and pozelimab, respectively, which are two anti-C5 mAbs currently in clinical development [13, 14]. Both mAbs blocked the hemolysis of antibody-sensitized chicken erythrocytes and bound to C5 noncompetitively with eculizumab. The noncompetitive nature of this binding is explained by the different binding epitopes on C5 for these antibodies. Thus, with reference to the domain designations of C5 [20], eculizumab interacts with the MG7 domain [10], crovalimab binds the MG1 domain [11] and pozelimab binds noncompetitively to sequences [12] that appear to fall within the MG6 domain. Inclusion of either TPP-2799 or TPP-3544 into mixtures of eculizumab and hC5 in various ratios in PBS buffer led to the formation of complexes that were much larger than the mass of complexes formed by a single bivalent antibody and two C5 molecules. Several complexes formed in compositions containing eculizumab in slight molar excess of a physiological concentration of C5 (400 nM), and TPP-2799 in concentrations ranging from equimolar to a fivefold excess over eculizumab, achieved sizes greater than 1500 kDa, which is consistent with the presence of up to four molecules of C5. With TPP-3544, complexes with molar masses greater than 1700 kDa were detected when equimolar with eculizumab, consistent with the presence of up to four molecules of C5, although the complexes were somewhat smaller at a fivefold excess of TPP-3544 over eculizumab. For both TPP-2799 and TPP-3544, patterns of complexes with similar retention times to those produced in PBS were also observed in the presence of more than 80% (v/v) human plasma using Alexa Fluor 488-labeled eculizumab. With the techniques employed, it is also not possible to determine whether the length and flexibility of some of the larger complexes might have allowed for cyclization by intramolecular association, as proposed for other antibody–antigen complexes [21, 22].

The present findings have implications for situations in which a recipient may be simultaneously exposed to more than one noncompeting, bivalent, anti-C5 mAb. For example, this could arise when a patient is converted from one such antibody to another in a clinical study under conditions in which the first antibody cannot be completely eliminated before administration of the second antibody because it is necessary to maintain complete pharmacodynamic suppression during the conversion. PNH presents such a scenario wherein the reappearance of even low levels of free C5 could result in an intravascular hemolytic crisis, particularly in patients with a large proportion of PNH red blood cells, or result in thrombosis owing to both intravascular hemolysis and complement activation of PNH white blood cells and platelets.

In parallel with our work in this and prior [23, 24] disclosures, others have reported on the formation of such complexes. Complexes between 1200 and 2100 kDa formed by pozelimab, C5, and their reagent grade eculizumab have been demonstrated *in vitro* in amounts representing about 12% of the total chromatographically recovered material [12].

Using a SEC and enzyme-linked immunosorbent assay (ELISA) [25], the mid to high molecular weight DTDCs in sera of PNH patients switched from eculizumab to crovalimab peaked at day 15 with complete elimination by day 78 [13]. The larger DTDCs, to elute, from SEC-ELISA appeared to constitute a significant amount of the initially administered dose [24], although an alternative dosing regimen in part 4 of the COMPOSER trial [26, 27] provided partial reduction in DTDCs in patients switched from eculizumab [26].

Immune complexes are taken up by cells of the reticuloendothelial system (RES) both through interaction of antibody Fc domains with fragment crystallizable gamma (Fcɤ) receptors and interaction of complement-dependent opsonins such as deposited C3 fragments with complement receptors and impairment of these processes can lead to tissue deposition and immune complex disease as reviewed elsewhere [28]. It is important therefore to understand whether large multivalent complexes such as those described presently might have an increased potential for tissue deposition because they comprise antibodies that have been Fc-engineered to eliminate or reduce their capacity to activate complement or bind to Fcɤ receptors [1, 11, 12].

Polyvalent interaction of immune complexes with neonatal Fc receptor, as reviewed by Baker et al. [29], is associated with augmented cell uptake, antigen processing, and induction of cellular immune responses. Together with the relatively high level of such complexes set by the abundance of circulatory C5 (approximately 75 μg/mL), this could raise the risk of immune reactivity against each antibody constituent within these multivalent complexes, potentially complicating not only the effectiveness of the new therapeutic, but also future use of the therapeutic being replaced.

Given the foregoing, a detailed characterization of the pharmacodynamic and pharmacokinetic properties of polyvalent complexes comprising noncompeting, bivalent, anti-C5 antibodies is warranted, as is the incorporation of mitigation processes to avoid the formation of these complexes in patients transitioning from therapy with one bivalent antibody to another. The formation of such complexes would be avoided by switching between antibodies recognizing the same epitope (e.g., eculizumab to ravulizumab) or by converting to monovalent anti-C5 development compounds.

The present findings raise potentially important general considerations prior to converting a patient from one IgG monoclonal antibody to a second antibody to the same target. This is particularly important as the available number of IgG based antibodies against a given target increases. Among the therapeutic monoclonal antibodies listed elsewhere as approved or in regulatory review [30], there are more than 10 different targets against which more than three different IgG based monoclonal antibodies are approved or in development, and this number is likely to increase. Before contemplating a switch from one antibody to another it is advised to consider: 1) whether the conversion features antibodies that are non-competing; 2) the degree to which the first antibody can be eliminated from the patient circulation prior to administration of the second antibody; 3) the latitude for selection of a dose of the second antibody that will disfavor the formation of large complexes and which in turn is a function of the therapeutic target level.

## Supporting information

S1 Fig Rayleigh ratios and molar masses of complexes formed between TPP-2799 and complement component C5 in phosphate-buffered saline. (A) TPP-2799 alone (700 nM). (B) TPP-2799 (700 nM) plus complement component C5 (400 nM). (C) complement component C5 alone (400 nM).
(EPS)

S2 Fig Rayleigh ratios and molar masses of complexes formed between TPP-3544 and complement component C5 in phosphate-buffered saline. (A) TPP-3544 alone (700 nM). (B) TPP-3544 (700 nM) plus complement component C5 (400 nM).
(EPS)

S3 Fig Rayleigh ratios and molar masses of ravulizumab alone (700 nM) in phosphate-buffered saline. (EPS)
